# Risk factors for suicidal tendency in people with epilepsy in China: a case–control study

**DOI:** 10.1038/s41598-021-81870-9

**Published:** 2021-02-02

**Authors:** Mintao Lin, Jiani Chen, Sisi Li, Yingjie Qin, Xuruan Wang, Yadong Liu, Ammar Taha Abdullah Abdulaziz, Wenyu Liu, Dong Zhou, Jinmei Li

**Affiliations:** 1 Department of Neurology, West China Hospital, Sichuan University, No. 37 Guoxue Road, Chengdu, 610041 Sichuan People’s Republic of China; 2 West China Medical School, Sichuan University, No. 37 Guoxue Road, Chengdu, 610041 Sichuan People’s Republic of China

**Keywords:** Epilepsy, Neurology

## Abstract

People with epilepsy (PWE) have an increased suicide prevalence. This study aimed to identify the risk factors for suicidal tendency among PWE in West China. A nested case–control study was designed in a cohort of patients with epilepsy (n = 2087). In total, 28 variates were calculated. In the univariate analysis, unemployment, low income, seizure frequency, seizure-free time, infectious or structural etiology, levetiracetam or phenobarbital use, anxiety, depression, and stigma were associated with suicidal tendency. A multivariate analysis indicated that unemployment (odds ratio [OR] 5.74, 95% confidence interval [CI] 2.13–15.48), levetiracetam use (OR 2.80, 95%CI 1.11–7.05), depression (C-NDDI-E score ≥ 13; OR 3.21, 95%CI 1.26–8.21), and stigma (SSCI score ≥ 16; OR 6.67, 95%CI 1.80–24.69) were independently associated with suicidal tendency. Conditional inference tree analysis indicated that SSCI and C-NDDI-E scores could effectively identify patients with suicidal tendency. Thus, this study suggests that unemployment, levetiracetam use, depression, and stigma are independent risk factors for suicidal tendency in PWE in China.

## Introduction

Epilepsy is a major neurological disorder that affects approximately nine million people in China^1^. It is associated with social distress, vulnerability, and reduced quality of life (QOL)^2^. In particular, comorbidities are associated with poor outcomes^3,4^, such as depression, which is the most common psychiatric comorbidity. Although suicide is more commonly reported among patients with antiepileptic drug (AED)-resistant epilepsy (25–55%)^5^, it can also occur in those with well-controlled seizures^5,6^. Suicide is also a cause of premature mortality in people with epilepsy (PWE)^7–9^. The proportion of death from suicide among PWE is 2.06–4.6 times higher than in the general population^10–12^.

The risk factors for suicide include being female, unemployment, low income, high seizure frequency, some AEDs, seizure-preceding auras, temporal epilepsy, psychiatric disorders, and antidepressant drugs^10,13,14^. The AEDs and psychiatric disorders have a larger influence on suicide than other risk factors. The use of new AEDs, including levetiracetam, topiramate, and vigabatrin, leads to a three-fold increase in the risk of suicide-related behavior when compared with no AED use^15^. The combination of three or more drugs is also associated with an increased risk of suicide^16^.

Other studies also found a connection between suicide and stigma^17^, as well as sleep quality^18^. Recent studies indicated that epilepsy, psychiatric disorders, and suicide may be pathophysiologically linked^19,20^. The risk of suicide was 2.6 times higher among PWE with psychiatric disorders, including major depression, anxiety, and psychosis, compared to those without mental illnesses^21^. Although many factors are associated with suicide in PWE, psychiatric illnesses and side effects of drugs are regarded as the most significant^10,22^.

To our knowledge, only one study has examined the factors associated with suicide among PWE in mainland China^23^. Considering the heavy burden and the serious consequences suicidality has on society, it is necessary and of interest to identify the clinical correlation of suicidal tendency in this population reveal the associated factors that may eventually lead to better control and prevention of suicide in PWE. Thus, we conducted this study using the database of the West China Hospital epilepsy center^24–26^.

## Methods

### Subjects

Our study was based on a database established in October 2014. The database enrolled adult patients with epilepsy who visited the West China Hospital for consultation between October 2014 and April 2019. Patients in the database were followed-up each year to update their clinical information. Their diagnosis of epilepsy was made by at least two board-certified professional neurologists. The inclusion criteria were as follows: (1) diagnosis of epilepsy according to the International League Against Epilepsy (ILAE) criteria, (2) willingness to provide information regarding the disease and their social situation, and (3) sufficient reading ability to understand the questionnaire.

Patients meeting any of the following criteria were excluded from this study: (1) under 18 years and (2) severe coexisting physical or psychiatric illnesses such as cancer, strokes, dementia, schizophrenia, or intellectual disability.

### Procedures

During the 2018 and 2019 follow-up, we evaluated all patients in the database using the Chinese version of the Neurological Disorders Depression Inventory for Epilepsy (C-NDDI-E), seven-item anxiety scale (GAD-7), Stigma Scale for Chronic Illnesses 8-item version (SSCI-8), and the Mini International Neuropsychiatric Interview (MINI). Once a moderate or high risk of suicide (suicidal tendency) was identified in a person with epilepsy, we randomly selected three patients without suicide risk from the study cohort, ensuring matching age (within 2 years) and gender.

For patients with a high risk of suicide, mental health support was offered by an experienced specialist. This study was approved by the Ethics Committee of West China Hospital of Sichuan University (Approval Number: hiCTR1900023087).

### Clinical information and sociodemographic status

All information was collected by experienced neurologists via face-to-face or telephonic interviews. Clinical factors including demographic information, age at disease onset, classification of seizure, surgery history, etiological factors, AED usage, electroencephalography, and imaging (magnetic resonance imaging/computed tomography) results of patients were all recorded in a standardized form by certified staff.

### Questionnaires

The MINI is a short structured diagnostic interview for psychiatric disordered classified using DSM-IV and ICD-10^27^. In this study, the risk of suicide was measured using the Suicidality Module of the MINI, which divides the risk of suicide into low (1–5), moderate (6–9), and high (≥ 10) risk. The validity of this scale (Chinese version 5.0.0) has been established in the Chinese population^28^. In this study, a high risk of suicide was defined as a MINI score ≥ 6. Notably, the MINI score indicates a low risk if there was no suicidal intention or ideation in the last month even if the patient had attempted suicide previously.

The C-NDDI-E is a reliable and valid screening tool for the detection of major depression in Chinese patients with epilepsy^29^. It is a six-item self-administered instrument with a total score ranging from 6 to 24. Each item is assigned a score of 1–4 (1 indicating “never,” and 4 indicating “always or often”). The optimal cut-off score for the C-NDDI-E was ≥ 13^29,30^.

The GAD-7 is used to screen for generalized anxiety disorder and assess its severity in clinical practice and research^31^. As each of the seven items is scored from 0 to 3, the GAD-7 score ranges from 0 to 21. Cut-off points of 5, 10, and 15 on the GAD-7 are interpreted as representing mild, moderate, and severe levels of anxiety, respectively^31^. The validity of this scale has been verified in the Chinese population^32^.

The SSCI-8 is a short form assessing enacted and internalized stigma for patients with neurological disorders^33^. The raw summed score range is 8–40 for this scale. The higher the score of a patient, the higher their level of stigma experienced. The validity of this scale has been established in the Chinese population^34^.

### Statistical analysis

Descriptive statistics of the patients and controls were conducted. Quantitative data were expressed as mean ± standard deviation, and qualitative data were summarized as proportions. Demographic and clinical data between the two groups were compared. Student’s t-test was used for continuous variables, and the chi-squared test was used for categorical variables.

To identify potential correlations between clinical or sociodemographic variables and suicidal tendency, a univariate analysis and conditional multiple logistic regression were performed. Crude odds ratios (ORs) were calculated based on the univariate analysis. The conditional multiple logistic regression model included (1) variables that yielded a *p*-value < 0.2 in the univariate analysis, and (2) clinical factors from a previous study that were associated with suicide (such as seizure type and frequency, aura, length of seizure-free time, age at onset, number of AEDs, and whether the patient experienced seizures while being awake). All analyses were conducted using SPSS 15.0 (SPSS Inc., Chicago, Illinois). All tests were two-tailed, and *p*-values < 0.05 were considered statistically significant.

Using R (R × 64 3.5, www.r-project.org) with the rattle package (5.2.0, rattle.togaware.com)^35^, a conditional inference decision tree was employed to recognize patients with suicidal tendency. To identify the many possible factors associated with suicidal tendency in PWE, the decision tree grows as more and more splits are defined, until it cannot find any predictor that leads to significantly different child nodes. The final sets of trees indicate the most effective associated factors.

## Results

**Figure 1:**
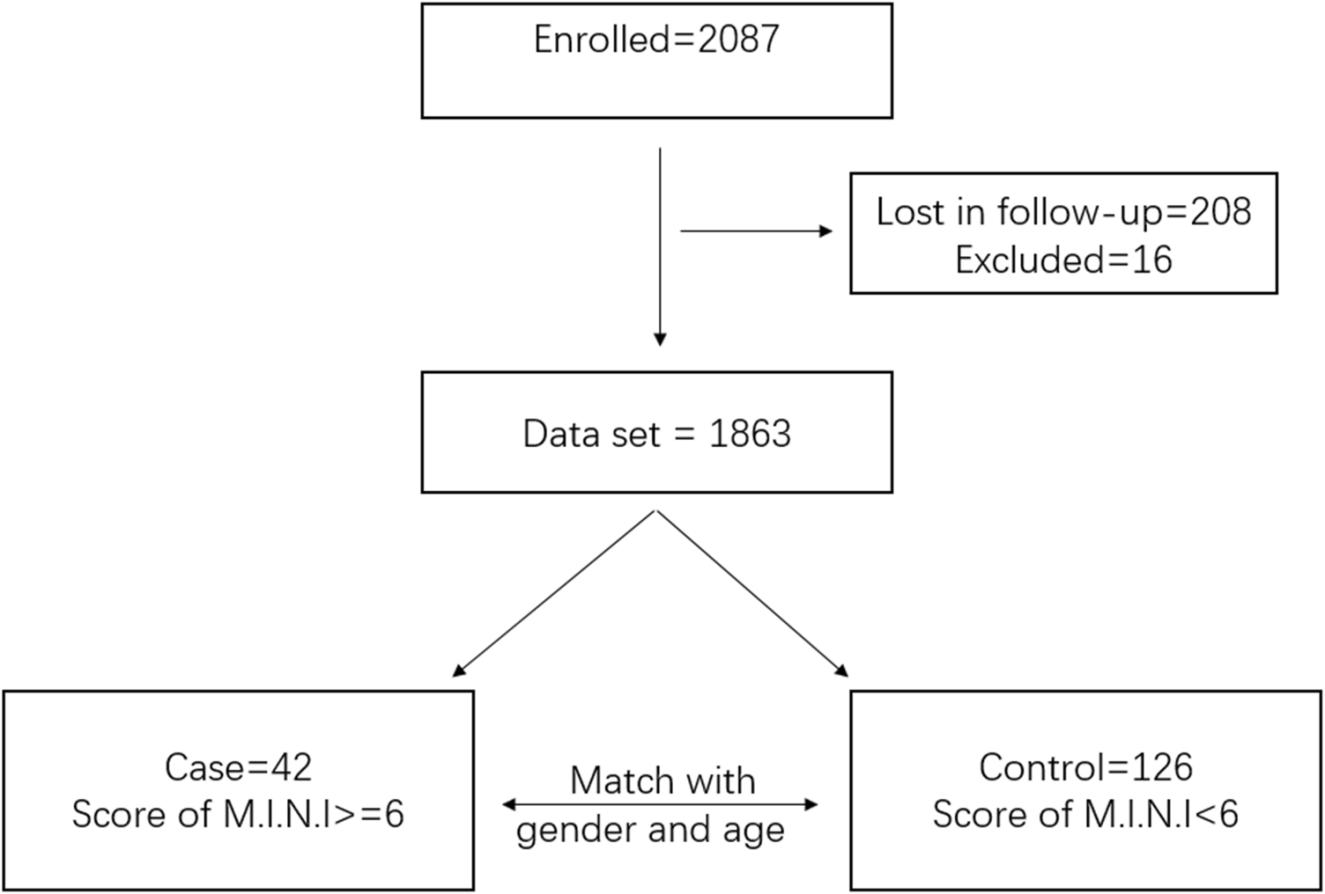
The flowchart of the study design.

A total of 2087 patients who visited the epilepsy clinic at the West China Hospital between October 2014 and April 2019 were invited to participate in the current study. In the 2018–2019 follow-up, 1879 patients agreed to continue participating in this study. Among them, 6 patients had a physical disability and 10 had a significant intellectual disability; thus, they were excluded and only 1863 patients completed the study. The descriptive statistics of the patients and controls are presented in Fig. 1.

A total of 42 people had suicidal tendencies (according to the MINI score), of which 40 had clear suicidal thoughts in the last month, 8 had attempted suicide, and 13 had suicide plans. Among the 8 patients who had attempted suicide, 6 had clear suicidal thoughts in the last month. The baseline demographic findings are shown in Table 1. There was no apparent difference between PWE with and without suicide risk in terms of average disease duration (10.64 ± 7.64 vs. 10.24 ± 7.67 years, p = 0.80) or mean age (27.55 ± 9.26 vs. 27.97 ± 9.38 years, *p* = 0.952).

**Table 1:** (a) Comparison of Baseline characteristics for cases and controls (b) Comparison of sociodemographic characteristics between PWE with high suicidal tendency and PWE without (Result of using univariable analysis).

Characteristics	With suicidal tendency N = 42	Controls N = 126	*P*	
*(a)*				
Gender (%)				
Male	16 (38.1)	48 (38.1)		
Female	26 (61.9)	78 (61.9)		
Mean age, years (SD)	27.55 (9.26)	27.97 (9.38)	0.95	
Mean disease duration (SD)	10.64 (7.64)	10.24 (7.67)	0.80	

Variable	With suicidal tendency (%) N = 42	Controls (%) N = 126	OR (95% CI)	*P*
*(b)*				
Marriage				
Single	26 (61.9)	76 (60.3)	1.0 (reference)	
Married	16 (38.1)	50 (39.7)	0.94 (0.46–1.92)	0.86
Child				
Have a child	32 (76.2)	85 (67.5)	1.0 (reference)	
Do not have a child	10(23.8)	41 (32.5)	0.65(0.29–1.45)	0.29
Living place				
City	27 (64.3)	92 (73.0)	1.0 (reference)	
Rural	15 (35.7)	34 (27.0)	1.50(0.72–3.16)	0.28
Education (years)				
≤ 6	4 (9.5)	4 (3.2)	1.0 (reference)	
7–9	9 (21.4)	24 (19.0)	0.38 (0.08–1.83)	0.23
10–12	16 (38.1)	40 (31.8)	0.40 (0.09–1.80)	0.23
13 +	13 (31.0)	58 (46.0)	0.22 (0.05–1.02)	0.05
Job				
Employment	13 (31.0)	72 (57.1)	1.0 (reference)	
Unemployment	24 (57.1)	26 (20.6)	5.11 (2.27–11.50)	0**
Retire	1 (2.4)	3 (2.4)	1.85 (0.18–19.15)	0.61
Student	4 (9.5)	25 (19.8)	0.89 (0.26–2.97)	0.85
Income (Yuan, per month)				
≥ 6000	9 (21.4)	31 (24.6)	1.0 (reference)	
4000–5999	5 (11.9)	34 (27.0)	0.51 (0.15–1.68)	0.27
2000–3999	9 (21.4)	40 (31.7)	0.78 (0.28–2.18)	0.63
< 2000	19 (45.2)	21 (16.7)	3.12 (1.18–8.20)	0.02*

*CI* confidence interval, *OR* odds ratio.

*P < 0.05; **P < 0.01.

The demographic and sociological associations are presented in Table 1. Unemployment (OR 5.11, 95% confidence interval [CI] 2.27–11.50) and low income (OR 3.12, 95% CI 1.18–8.20) were significantly different between the two groups.

The clinical characteristics of the patients are shown in Table 2. The case and control groups were not significantly different in terms of age at onset, aura, non-psychiatric comorbidities, and seizure type. Compared to those with 0–3 seizures per year, patients experiencing seizures on a weekly basis presented a higher risk of suicide (OR 2.71, 95% CI 1.10–6.65). Compared to patients without any seizure in the past 12 months, patients with recurrent seizures during the past 3 months had an increased odds ratio of suicide (OR 5.86, 95% CI 1.67–20.59). Among etiological factors, infectious increased the risk of suicide (OR 4.71, 95% CI 1.35–16.37), and a structural etiology was associated with a higher odds ratio (OR 2.58, 95% CI 1.17–5.69) than an unknown etiology.

**Table 2:** Comparison of clinical characteristics between PWE with high suicidal tendency and PWE without (Result of using univariable analysis).

Variable	With Suicidal Tendency (%) N = 42	Controls (%) N = 126	OR (95%CI)	*P*
**Age at onset of epilepsy(years)**				
30 +	5 (11.9)	12 (9.5)	1.0 (reference)	
18–30	11 (26.2)	41 (32.5)	0.64 (0.19–2.21)	0.49
10–18	18 (42.9)	59 (46.8)	0.73 (0.23–2.35)	0.60
< 10	8 (19.0)	14 (11.1)	1.37 (0.35–5.33)	0.65
**Frequency (per year)**				
0–3	11 (26.2)	66 (52.4)	1.0 (reference)	
Yearly	8 (19.0)	18 (14.3)	2.67 (0.93–7.62)	0.07
Monthly	14 (33.3)	31 (24.6)	2.71 (1.10–6.65)	0.03*
Weekly	9 (21.4)	11 (8.7)	4.91 (1.65–14.57)	< 0.01**
**No-seizure time(months)**^**a**^				
> 12	3 (7.1)	34 (27.0)	1.0 (reference)	
6–12	4 (9.5)	19 (15.1)	2.39 (0.48–11.80)	0.29
3–6	4 (9.5)	13 (10.3)	3.49 (0.69–17.76)	0.13
< 3	31 (73.8)	60 (47.6)	5.86 (1.67–20.59)	0.01**
*Aura*				
No	25 (59.5)	83 (65.9)	1.0 (reference)	
Yes	17 (40.5)	43 (34.1)	1.31 (0.64–2.69)	0.46
**Nonpsychiatric comorbidities**				
No	37 (88.1)	116 (92.1)	1.0 (reference)	
Yes	5 (11.9)	10 (7.9)	1.56 (0.50–4.88)	0.44
**Etiology**				
Unknown	17 (40.5)	80 (63.5)	1.0 (reference)	
Genetic	1 (2.4)	8 (6.3)	0.59 (0.07–5.02)	0.63
Immune	1 (2.4)	16 (0.8)	4.71 (0.28–79.01)	0.28
Infectious	6 (14.3)	31 (4.8)	4.71 (1.35–16.37)	0.02*
Structural	17 (40.5)	80 (24.6)	2.58 (1.17–5.69)	0.02*
**Types**				
Focal	34 (81.0)	88 (69.8)	1.0 (reference)	
Generalized	8 (19.0)	38 (30.2)	0.55 (0.23–1.29)	0.17

^a^No-seizure time: The time(month) without seizure occurring.

*CI* confidence interval, *OR* odds ratio.

*P < 0.05; **P < 0.01.

The use of antiepileptic drugs for the two groups is compared in Table 3. In comparison with a single drug, three or more drugs were associated with an increased suicidal tendency (OR 4.36, 95% CI 1.56–12.18). Regarding individual AEDs, using levetiracetam (OR 3.76, 95% CI 1.73–8.14) and phenobarbital (OR 5.54, 95% CI 1.26–24.29) were associated with suicidal tendency.

**Table 3:** Comparison of the use of antiepileptic drugs between PWE with high suicidal tendency and PWE without (Result of using univariable analysis).

Variable	With Suicidal Tendency (%) N = 42	Controls (%) N = 126	OR (95%CI)	*P*
**Time of AEDs (months)**				
≤ 24	12 (28.6)	30 (23.8)	1.0 (reference)	
25–48	6 (14.3)	22 (17.4)	0.68 (0.22–2.10)	0.50
49–84	11 (26.1)	28 (22.2)	0.98 (0.37–2.58)	0.97
85–120	3 (7.1)	26 (20.6)	0.29 (0.07–1.14)	0.08
121 +	10 (23.8)	20 (15.9)	1.25 (0.45–3.44)	0.67
**Levetiracetam**				
No	11 (26.2)	72 (57.1)	1.0 (reference)	
Yes	31 (73.8)	54 (42.9)	3.76 (1.73–8.14)	< 0.01**
**Valproate**				
No	27 (64.3)	79 (62.7)	1.0 (reference)	
Yes	15 (35.7)	47 (37.3)	0.93 (0.45–1.93)	0.85
**Topiramate**				
No	37 (88.1)	119 (94.4)	1.0 (reference)	
Yes	5 (11.9)	7 (5.6)	2.30 (0.69–7.67)	0.18
**Lamotrigine**				
No	30 (71.4)	95 (75.4)	1.0 (reference)	
Yes	12(28.6)	31 (24.6)	1.23 (0.56–2.68)	0.61
**Oxcarbazepine**				
No	31 (73.8)	88 (69.8)	1.0 (reference)	
Yes	11 (26.2)	38 (30.2)	0.82 (0.37–1.80)	0.62
**Carbamazepine**				
No	37 (88.1)	112 (88.9)	1.0 (reference)	
Yes	5 (11.9)	14 (11.1)	1.08 (0.37–3.21)	0.89
**Carbamazepine**				
No	41 (97.6)	126 (100)	1.0 (reference)	
Yes	1 (2.4)	0(0)	NA	NA
**Phenobarbital**				
No	37 (88.1)	123 (97.6)	1.0 (reference)	
Yes	5 (11.9)	3 (2.4)	5.54 (1.26–24.29)	0.02*
**Phenytoin**				
No	42 (100)	123 (97.6)	1.0 (reference)	
Yes	0 (0)	3 (2.4)	NA	NA
**No. of AEDs**				
1	13(31.0)	68(54.0)	1.0 (reference)	
2	19 (45.2)	46 (36.5)	2.16 (0.97–4.80)	0.06
≥ 3	10 (23.8)	12 (9.5)	4.36 (1.56–12.18)	0.01**
**Side effect**				
No	31 (73.8)	99 (78.6)	1.0 (reference)	
Yes	11 (26.2)	27 (21.4)	1.30 (0.58–2.92)	0.52

*CI* confidence interval, *OR* odds ratio.

*P < 0.05; **P < 0.01.

According to the questionnaire scores, presence of high anxiety (GAD-7 ≥ 15; OR 4.02, 95%CI 1.10–14.70), depression (C-NDDI-E ≥ 13; OR 5.83, 95%CI 2.70–12.61), and stigma (SSCI ≥ 16; OR 6.98, 95%CI 2.45–19.88) were all associated with suicidal tendency in PWE. This is depicted in Table 4.

**Table 4:** (a) Comparison of GAD-7, C-NDDI-E and SSCI between PWE with high suicidal risk and PWE without (using univariable regression) (b) Multivariate conditional logistic regression analysis of association factors for suicidal tendency.

Variable	With Suicidal Tendency (%) N = 42	Controls (%) N = 126	OR (95%CI)	*P*
*(a)*				
**GAD-7**				
< 5	17 (40.5)	82 (65.1)	1.0 (reference)	
5–9	19 (45.2)	35 (27.8)	2.62 (1.22–5.63)	0.01*
10–14	1 (2.4)	3 (2.4)	1.61 (0.16–16.41)	0.69
15 +	5 (11.9)	6 (4.8)	4.02 (1.10–14.70)	0.04*
**C-NDDI-E**				
≤ 12	13 (31.0)	98 (77.8)	1.0 (reference)	
13 +	29 (69.0)	28 (22.2)	7.81 (3.59–16.99)	< 0.01**
**SSCI**				
≤ 8	6 (14.3)	40 (31.7)	1.0 (reference)	
9–15	14 (33.3)	65 (51.6)	1.44 (0.51–4.04)	0.49
16 +	22 (52.4)	21 (16.7)	6.98 (2.45–19.88)	< 0.01**

Variable	OR (95% CI)	*P*		
*(b)*				
**Job**				
Yes	1.0 (reference)			
None	5.74 (2.13–15.48)	< 0.01**		
Retire	5.02 (0.41–61.76)	0.21		
Student	0.73 (0.18–2.88)	0.65		
**LEV**				
No	1.0 (reference)			
Yes	2.80 (1.11–7.05)	0.03		
**C-NDDI-E**				
< 13	1.0 (reference)			
14 +	3.21 (1.26–8.21)	< 0.01**		
**SSCI**				
≤ 8	1.0 (reference)			
9–15	1.48 (0.45–4.81)	0.52		
16 +	6.67 (1.80–24.69)	< 0.01**		

*GAD-7* seven-item anxiety scale, *C-NDDI-E* Chinese version of the Neurological Disorders Depression Inventory for Epilepsy, *SSCI-8* The Stigma Scale for Chronic Illnesses 8-item version,* LEV* levetiracetam, *CI* confidence interval, *CI* confidence interval, *OR* odds ratio.

*P < 0.05; **P < 0.01.

Multivariate logistic regression analysis (see Table 4) revealed that unemployment (OR 5.740, 95% CI 2.13–15.48), use of levetiracetam (OR 2.80, 95% CI 1.11–7.05), depression (C-NDDI-E score ≥ 13; OR 3.21, 95% CI 1.26–8.21), and stigma (SSCI-8 score ≥ 16; OR 6.68, 95% CI 1.80–24.69) were independently associated with an increased suicide score ( score of the MINI ≥ 6).

**Figure 2:**
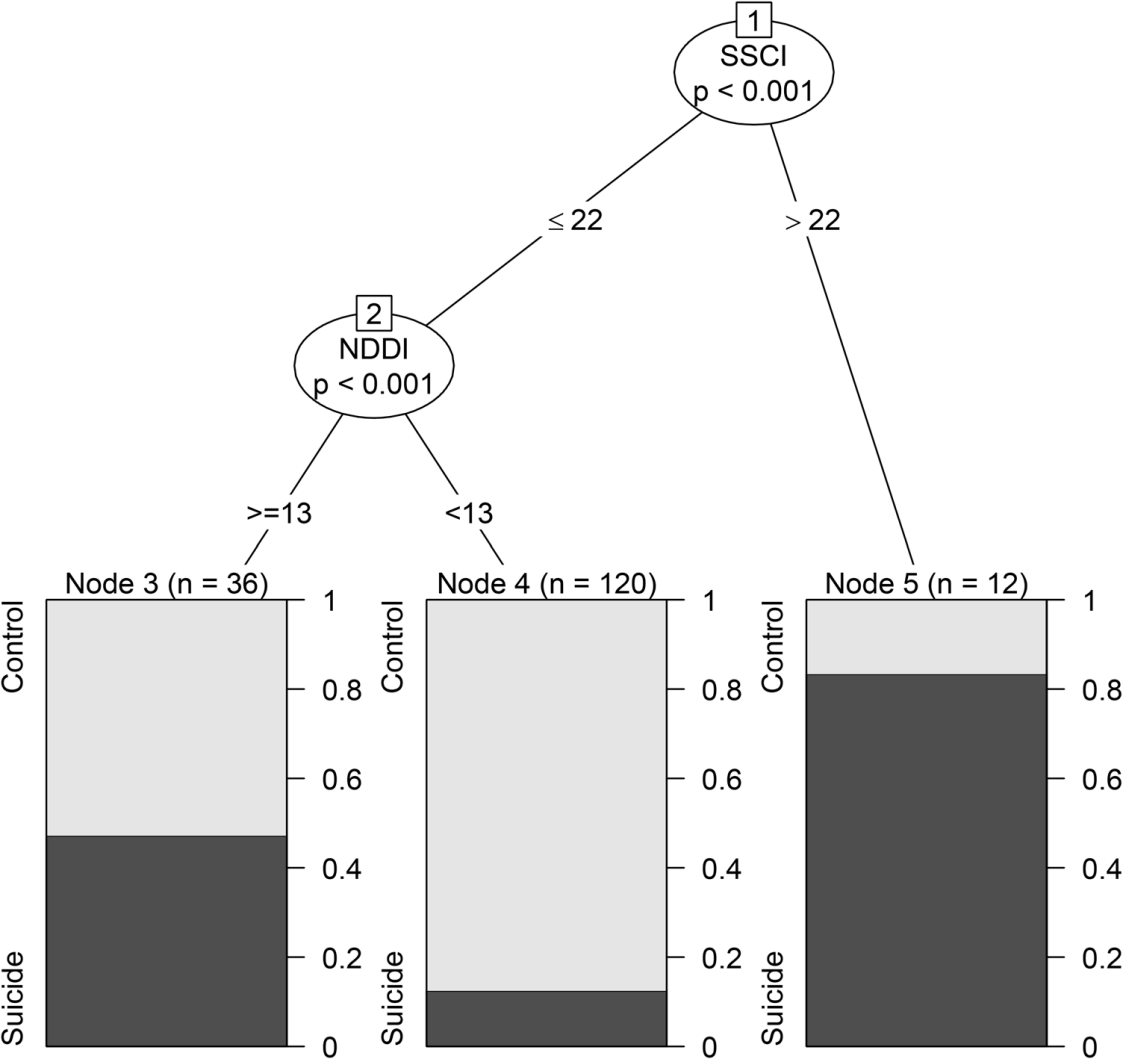
The Result of conditional inference tree: It shows the most significant factors were SSCI and NDDI(Using R × 64 3.5, www.r-project.org); Two examples to show how to use it: Patient 1 got 20 scores in SSCI and 14 scores in NDDI, then he was divided into Node 3, which means he has about 48% possibility to have suicidal tendency. Patient 2 got 24 scores in SSCI, then he was divided in node 5, which means he has more than 80% possibility to have suicidal tendency.

The conditional inference tree in Fig. 2, shows that SSCI and C-NDDI-E scores could effectively estimate whether a patient had suicidal tendency. Of the 29 predictors, the conditional inference tree analysis identified the SSCI and C-NDDI-E scores as significant risk factors that could discriminate between the two groups. Based on these factors, individuals were stratified into three groups: (1) SSCI ≤ 22 and C-NDDI-E < 13 (12.5% of the participants with suicidal tendency), (2) SSCI ≤ 22 and C-NDDI-E ≥ 13 (46.7% of participants with suicidal tendency), and (3) SSCI > 22 (83.3% of participants with suicidal tendency).

## Discussion

Compared to other areas in China, the southwestern region is a relatively underdeveloped area. To date, many studies have examined the risk factors that are associated with suicidal tendency among PWE in developed areas or countries, but few studies have enrolled populations of underdeveloped areas. Our study focuses on various association factors in this population, which may differ from those in developed regions. We found a 2.3% the incidence of suicide while the rates of suicide differ across other regions. For instance, in Korea, suicidality was present in 208 (30.4%) of the 684 PWE^10^. In Taiwan, the overall incidence rate of suicide attempts among PWE was 15.7 per 100,000 people per year. Although a study in China estimated the risk factors of suicidal tendency, it did not provide their rate of suicidal tendency^23^. We look forward to more reports from the west of China to compare the results. Meanwhile, compared with healthy people in China, the rate of suicidal tendency among PWE is higher (2.3% vs. 0.8%)^36^, which is consistent with other studies^18,22,23^.

Our results demonstrate that unemployment and low income play significant roles in the assessment of suicidal tendency in PWE. A previous study suggested a strong relationship between socioeconomic factors or poverty and suicide attempts in patients with chronic disorders^37^. To improve patients’ life span and QOL, an optimal interaction between PWE and the social environment is needed. This interaction could be obtained through long-term health-related support. Past research shows that PWE have a higher economic burden compared to healthy people in China^38^. Considering the risk of suicide in unemployed and low-income patients, we should consider their economic burden during diagnosis and treatment. When necessary, healthcare professionals could also refer these patients to employment counseling. In addition, unemployment is a risk factor in the general population. In a Hong Kong study^39^, the OR for unemployment was 9.40 (95% CI 4.94–17.88), while in our study, it was 5.11 (95% CI 2.27–11.50). As a result, since there is no statistical difference, we cannot conclude that unemployment is a greater risk for suicide for PWE than it is for healthy people.

In our study, depression and anxiety were highly related to suicidal behavior, which is consistent with the findings of previous studies. Meanwhile, our study showed that stigma also plays an important role. A study reported that stigmatization is correlated with low self-esteem and self-competence, a lowered sense of capability, an increased sense of vulnerability, increased rates of both depression and anxiety symptoms, and, ultimately, decreased life satisfaction^40^. Both personal and external factors may have a negative impact on interpersonal relationships, including lower rates of schooling, employment, leisure activities, marriage, driving, and other types of social interaction, which may result in social isolation, discrimination, and misconception. These may have adverse effects on QOL^41^. It is well known that epilepsy is associated with high levels of stigma^17,40^. Epilepsy is commonly misunderstood as a disorder that represents a form of “insanity” or “madness,” thus harming the reputation of PWE^42–44^. It is necessary to improve public health education and reduce discrimination against these patients. Meanwhile, medical professionals should employ more screenings for stigma during the diagnosis and treatment of suicidal ideation.

In the present study, using levetiracetam was independently associated with suicidal tendency, which is consistent with the results of published studies^8,22^. A previous study^15^ found that the use of newer AEDs (e.g., levetiracetam, tiagabine, topiramate, and vigabatrin), which have a high potential for causing depression^12^, is associated with a threefold increased risk of self-harm/suicidal behavior when compared to other treatment options. In our study, levetiracetam was associated with a 3.76-fold (95%CI 1.73–8.14) of suicidal tendency, which is similar to the value reported by Andersohn et al.^15^. Phenobarbital was also associated with a higher suicide risk in the current study. Interestingly, phenobarbital has not previously been reported to be associated with an increased risk of suicide among PWE, whereas in the current study, this risk was 5.54-fold (95%CI 1.26–24.28). This finding is important because phenobarbital is still more widely used in rural China^45^ than in developed countries. Therefore, healthcare professionals in developing countries should be encouraged to closely assess and monitor the patients using phenobarbital for possible suicidal tendencies.

In our study, which is based on the 2017 classification of ILAE^46^, infectious and structural etiologies were associated with suicidal tendency in PWE, while previous studies were based on the 1989 classification^47,48^.

The results of the conditional inference tree analysis suggest that SSCI and C-NDDI-E scores are useful for the identification of PWE with suicidal tendency. They indicate that if SSCI and C-NDDI-E scores are relatively high, then healthcare professionals should pay more attention to the suicide risks in such patients; for them, healthcare professionals should avoid prescribing AEDs that may increase the risk of suicide. In a future study, we will combine SSCI and C-NDDI-E scores to assess suicidal tendency because of the result of conditional inference tree analysis.

### Limitation

The present study has some limitations: (1) As a single-center study, patients were recruited at a tertiary referral center. Recruitment from such a setting may have introduced some selection bias toward more severe cases of epilepsy. In the future, we intend to collaborate with many epilepsy centers in other areas to avoid this possible bias. (2) The sample of patients with suicidal behavior was too small for further investigation. Although there were more than 1800 participants in the sample pool, less than 50 participants had a high risk of suicide. Owing to the limited sample size, the confidence intervals were wider. The model conditional inference tree analysis might be less effective. For future research, a longitudinal design is needed to identify changes in suicidal behaviors that may occur at different stages of epilepsy.

## Conclusion

This study suggests that unemployment, levetiracetam use, depression, and stigma are independent risk factors for suicidal tendency in PWE in China.

### Ethical standards

The study was approved by the Ethics Committee of the West China Hospital of Sichuan University as part of a large outpatient registration and follow-up project (approval number hiCTR1900023087). All patients provided written informed consent prior to their participation. This study was conducted in accordance with the ethical guidelines of the Declaration of Helsinki.
